# Perception of body movement when real and simulated displacements are combined

**DOI:** 10.1371/journal.pone.0193174

**Published:** 2018-03-06

**Authors:** Sébastien Caudron, Hadrien Ceyte, Pierre-Alain Barraud, Corinne Cian, Michel Guerraz

**Affiliations:** 1 Université de Lorraine, EA 3450 DevAH-Development, Adaptation & Disability, Vandoeuvre-lès-Nancy, France; 2 Univ. Grenoble Alpes, CNRS, CHU Grenoble Alpes, Grenoble INP, TIMC-IMAG, Grenoble, France; 3 Institut de Recherche Biomédicale des Armées, Brétigny sur Orge, France; 4 Univ. Grenoble Alpes, Univ. Savoie Mont Blanc, CNRS, LPNC, Grenoble, France; Ludwig-Maximilians-Universitat Munchen, GERMANY

## Abstract

Muscle-tendon vibration has often been used to study the contribution of proprioception to kinesthesia and postural control. This technique is known to simulate the lengthening of the vibrated muscle and, in the presence of balance constraints, evoke compensatory postural responses. The objective of the present study was to clarify the consequences of this stimulation on the dynamic features of whole-body movement perception in upright stance and in the absence of balance constraints. Eleven participants were restrained in a dark room on a motorized backboard that was able to tilt the upright body around the ankle joints. The participants were passively tilted backwards or forwards with a maximum amplitude of four degrees and at very low acceleration (thus preventing the semicircular canals from contributing to movement perception). In half the trials, the body displacement was combined with continuous vibration of the Achilles tendons, which simulates a forward tilt. Participants used a joystick to report when and in which direction they perceived their own whole-body movement. Our results showed that during backward whole-body displacement, the movement detection threshold (i.e. the minimum angular velocity required to accurately perceive passive displacement) was higher in the presence of vibration, whereas the accuracy rate (i.e. the proportion of the overall trial duration during which the movement was correctly indicated) was lower. Conversely, the accuracy rate for forward displacements was higher in the presence of vibration. In the absence of vibration, forward movement was detected earlier than backward movement. The simulated whole-body displacement evoked by Achilles tendon vibration was therefore able to either enhance or disrupt the perception of real, slow, whole-body tilt movements, depending on the congruence between the direction of real and simulated displacements.

## Introduction

Although most animal species move through their environment, human beings are greatly accustomed to being moved passively from one point to another (in vehicles or elevators, for example). Therefore, the accurate detection of (and distinction between) movements of the environment, one’s own movements in this environment, and changes in the position of one body segment (relative to other segments) are crucial in everyday life. This kinesthetic perception, which requires the sensing of body or limb positions and movements [1], is built by integrating visual [2–4], vestibular [5,6], and/or somatosensory inputs, particularly the sensory information provided by musculotendinous receptors [7–9]. The sense of movement is especially important for postural control: maintaining balance during stance requires the accurate detection of body sway, such as whole-body tilt around the ankle joints (in a simplified model of static postural control [10]). Fitzpatrick & McCloskey [11] showed that when vestibular, visual and peripheral proprioceptive sensory inputs are available, a human being is able to perceive anteroposterior body sway very sensitively—typically with as little as 0.06 to 0.12 degrees of rotation at the ankles. The latter researchers found that this threshold did not change as a function of vision availability or the forward vs. backward tilt of the body. Moreover, the threshold for movement detection fell as the velocity of body sway increased. Interestingly, Fitzpatrick & McCloskey [11] also found that the thresholds for the perception of body sway during standing were similar when (i) sensory input was limited to proprioceptive input from the leg and (ii) all sensory inputs were available. This finding indicates that somatosensory input and, in particular, muscle proprioception contribute predominantly to the accurate perception of body movement—especially in standing position [12–14].

The application of vibratory stimulation to either a muscle belly or a tendon is commonly used to investigate the role of proprioception input both in kinesthesia and in postural control, via a representation of the body’s static and dynamic geometries [15–16]. In the 80–100 Hz range, the muscle spindles and especially the primary endings respond maximally to the vibration. When the perceptual consequences are considered, muscle-tendon vibration can elicit kinesthetic illusions: the central nervous system seems to interpret the afferent discharges (mainly from type Ia sensory fibers) as resulting from lengthening of the vibrated muscle [8,17,18]. The vibratory stimulations are therefore often accompanied by illusory sensations of joint displacements or changes in body segment position [7–9,19]. This phenomenon has been widely described for the upper limb, in particular by means of position-matching tasks that compare a vibrated reference arm and an indicator arm [2,7,20–22]. For example, vibration of the elbow flexors in the absence of vision often leads to the kinesthetic illusion of extension of the indicator arm. When this same stimulation also produces true flexion of the vibrated arm (due to a tonic vibratory reflex), the latter is either less accurately perceived, not perceived at all, or perceived as occurring in the opposite direction (i.e. extension) [7,22]. In addition to these kinesthetic effects, muscle vibration also induces some other perceptual effects–emphasizing that the integration of proprioceptive input from the vibrated muscle contributes to a broader reorganization of space perception. For example, unilateral neck muscle vibration may induce visual illusions (illusory displacement of a small, luminous, static target) [23] and modifications of the egocentric space perception (deviation of the visual subjective straight-ahead toward the vibrated side) [9]. The fact that kinesthetic illusions of whole-body tilt have been also reported upon vibration of the lower limbs [24] raises the question of how the vibration (i.e. simulated lengthening) of postural muscles can modify a person’s perception of his/her whole-body orientation into its static and dynamic component parts.

According to Massion [25], postural control serves in fact two main functions: (i) a mechanical antigravity function, in which “equilibrium” (balance) is the key concept, and (ii) an interaction function in which the “body orientation” serves as a reference frame for the perception of and action on the outside world. Thus, postural control combines spatial orientation of the body with balance control. Given that muscle-tendon vibration is also an excellent means of inducing specific proprioceptive disturbances on “equilibrium”, its impact of this vibration on postural balance control has been extensively described in the literature; indeed, centrally modulated compensatory postural readjustments are induced in response to the vibratory stimulation of different muscles along the postural chain [16,26–30]. More specifically, application of vibration to the *tibialis anterior* muscles results in forward body sway, whereas a stimulation of the *triceps surae* muscles causes backward body sway [15]. Although it has been hypothesized that the vibration-induced simulated lengthening of a postural muscle is directly associated with the subsequent motor postural response (i.e. a displacement in direction of the vibrated muscle), the potentially causal nature of this relationship has never been unambiguously determined. In contrast, the impact of vibratory stimulations on whole body orientation has been less studied—partly because this function must be investigated in the absence of balance constraints. To overcome this challenge, Ceyte et al. [31] restrained blindfolded participants in an upright position on a servo-controlled, motorized backboard that was able to rotate in the sagittal plane around the ankles. The device therefore placed the participant in a particular orientation before he/she attempted to return to a vertical position. During bilateral vibration of the Achilles tendons, participants reported a subjective vertical orientation when their body was positioned with a backward tilt. This outcome might have been due to a vibration-induced perception of a simulated, forward movement.

As much as subjective vertical orientation, perception of body movement is a key component of postural regulation. As for the former, studying the impact of vibratory stimulation on the perception of body movement *per se* requires the participant to be moved back and forth (for instance), but above all in the absence of balance constraints. To the best of our knowledge, this situation has not previously been investigated. Hence, the objective of the present study was therefore to extend Ceyte et al.’s work [31] to perception of body movement around the ankles—i.e. the kinesthetic component of postural orientation. Participants were freed of postural constraints, and were passively tilted forwards or backwards in the sagittal plane. At the same time, Achilles tendon vibration was applied to simulate lengthening of the *triceps surae* and thus forward whole-body tilt. The perception of forward / backward whole-body movement was studied when this simulated displacement was combined with a real congruent (forward) or incongruent (backward) body displacement. If Achilles tendon vibration is involved in the perception of body movement around the ankle, one would expect Achilles tendon vibration to be associated with better perception of forward, passive, whole-body movements and, on the contrary, worse perception of backward movements.

## Method

### Participants

Eleven healthy adult participants (mean ± standard deviation age: 29 ± 4.3 years; 6 women) with no history of balance or neuromuscular disorders took part in the experiment after they had given their written, informed consent. The study was approved by the local independent ethics committee (CPP Sud-Est, Centre Hospitalier Universitaire de Grenoble, Grenoble, France; reference number: CPP08-CRSS-01) and performed in accordance with the ethical standards specified by the 1964 Declaration of Helsinki and subsequent revisions.

### Apparatus

#### The servo-controlled, motorized backboard (passive displacement)

**Fig 1:**
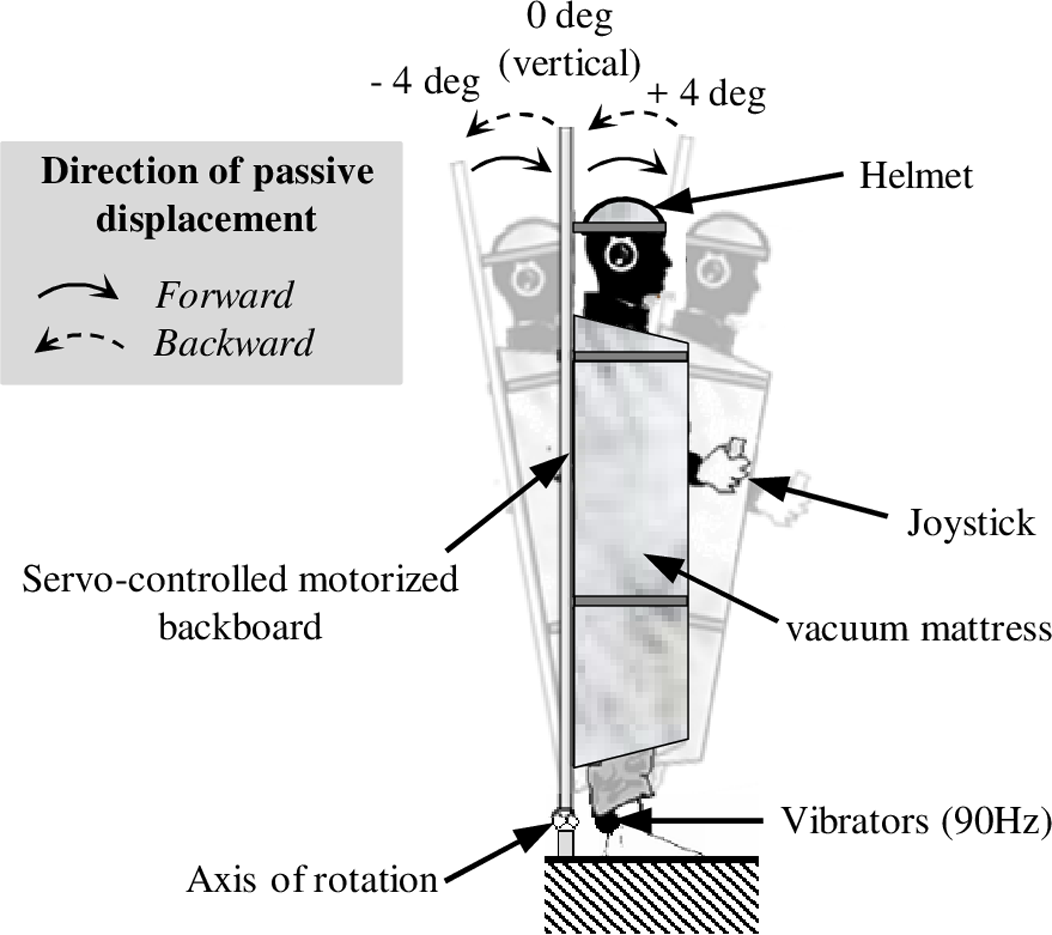
The experimental setup. Participants were restrained in the dark on a servo-controlled, motorized backboard able to tilt them around the ankle joints. They were passively tilted (total amplitude of each displacement: 4 deg) backwards or forwards. Two inertial vibrators were attached to the ankles. Participants used a joystick to report the perceived duration and direction of the whole-body movement.

**Fig 2:**
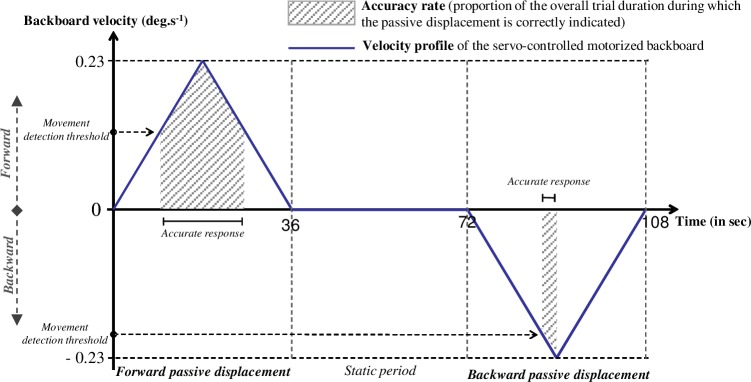
Examples of forward (upward) and backward (downward) passive displacements of the backboard, and the computed dependent variables. The backboard’s velocity profile (with the board tilted at a constant acceleration or deceleration of 0.0125 deg.s^−2^) is indicated by the blue line. Periods that were accurately perceived by the participant are indicated by the hatched area. The movement detection threshold (in deg.s^-1^) was determined for each trial as the instantaneous angular velocity recorded at the time of the first accurate response maintained for at least 300 ms. Note that, in VIB condition, vibrators were switched on throughout the entire block.

In order to move the whole body passively, participants were restrained on a motorized backboard (Fig 1) that could be rotated in the sagittal plane (pitch). The backboard was moved by an electrical low noise and vibration servomotor (MA-30, Mavilor Motors SA, Barcelona, Spain) using an epicycloïdal gear reducer (Servo-gearboxes SRP, REDEX, Ferrières, France), in order to transmit torques to the backboard, and a closed-loop servomechanism to control its trajectory. During passive displacements, the backboard was tilted forward or backward with a maximum angular velocity of 0.23 deg.s^−1^ (Fig 2). The initial acceleration for reaching this maximal velocity and the final deceleration for returning to a static position were set to a constant value of 0.0125 deg.s^−2^. Tilting the backboard at this very low acceleration prevented the semicircular canals from contributing to the perception of movement [32]. The pivot on which the platform rotated was situated at the level of the malleoli. Thus, rotation of the backboard tilted the participant’s whole body by four degrees backwards or forwards, while the soles of the feet remained parallel to the ground. This range of amplitudes was chosen because it corresponds to the sway that a standing participant can produce without losing his/her balance. A 36s period of displacement was necessary to reach this amplitude. The backboard’s angular position (accuracy: 5×10^−4^ deg) was controlled and recorded using a digital optical position encoder with a sample frequency of 10 Hz. The backboard’s displacement sequences were controlled by a computer.

#### Vibratory stimulation

Two inertial vibrators (VB115, Techno Concept, Mane, France) were attached to the ankles with elastic straps, so as to apply vibration bilaterally to the Achilles tendons. The vibration amplitude (200 μm) and frequency (90 Hz) were optimal for producing type Ia fiber responses.

### Procedure

Once the participant had adopted a comfortable upright position, he/she was secured to the backboard by means of three large straps (around the forehead, thorax and thighs, respectively). The strap system stabilized the participant and thus removed balance constraints. The participants were wrapped in a full-body vacuum mattress and wore a helmet, so that artificial tactile stimulations (induced by these devices) were homogeneously disseminated. Therefore, it minimized tactile somesthetic cues due to straps pressure. Participants were isolated from the vibration and from the backboard motor’s noise by headphones, which broadcasted pink noise. The experiment took place in a completely dark room, and participants were told to keep their eyes closed throughout the experimental sessions.

Participants used a single-axis joystick to report in real time when and in which direction (forward vs. backward) they perceived their own whole-body tilt: they had to push and hold the joystick in the perceived direction of movement and release it only when they perceived themselves to be static. The servo-controlled, motorized backboard produced passive, whole-body tilting displacement with an amplitude of four degrees in a backward direction (Bw: +4 deg to 0 deg, or 0 deg to -4 deg) or in a forward direction (Fw: -4 deg to 0 deg and 0 deg to +4 deg) (Fig 1). After the tilting displacement, the backboard could be immobilized, either in a vertical (0 deg), in a forward (+4 deg) or in a backward (-4 deg) position during 36 seconds (i.e. the length of the trial with a passive tilt). There were two experimental sessions, each of which comprised three trials in each passive tilt displacement condition. The passive displacement trials were presented in pseudo-random order and mixed with static trials. Each session was performed with continuous Achilles tendon vibration [VIB] or without vibration [NO VIB], and was divided in two blocks. The order of the four blocks was randomized, and a minimal 2 minute rest period was inserted between blocks. Each block started with approximately 1 minute period during which participants were in the vertical position. In VIB condition, vibrators were switched on throughout the entire block including the 1 minute period in the vertical position.

### Data processing and statistical analysis

The joystick position (backward, neutral or forward) was recorded at a frequency of 10 Hz and synchronized with the backboard recordings by using the digital position encoder. During a given trial, participants could therefore indicate backward movement, forward movement or no movement (Fig 2). The accuracy rate was defined as the proportion (in %) of the overall trial duration during which the passive displacement was correctly indicated by the participant. Moreover, the backboard’s angular velocity was derived from the backboard position recordings and then filtered with a second-order, low-pass Butterworth filter (cut-off: 1 Hz). The movement detection threshold (in deg.s^-1^) was determined for each trial as the instantaneous angular velocity recorded at the time of the first accurate response maintained for at least 300 ms. For movement detection threshold, only trials during which an accurate response was provided (at least for a given period) were considered.

Quantitative data were expressed as the mean (m) ± standard deviations (SD). For the accuracy rate and the movement detection threshold, differences between body orientations during the backward displacements (+4 deg to 0 deg vs. 0 deg to -4 deg) in one hand, and during the forward displacements (-4 deg to 0 deg vs. 0 deg to +4 deg) in the other hand, were compared using pairwise Student’s t-tests for each experimental condition (Bw-VIB, Bw-NO VIB, Fw-VIB, and Fw-NO VIB). The accuracy rate and the movement detection threshold were analyzed with separate 2×2 analyses of variance (ANOVAs) with repeated measures for the two factors “direction of displacement” [Bw *vs*. Fw] and “vibration condition” [VIB *vs*. NO VIB]. We checked that the conditions for performing an ANOVA (a normal data distribution) were met by applying a Shapiro-Wilk test to all the experimental conditions. The Newman–Keuls method (NK) was used for post hoc comparisons, whenever necessary. The threshold for statistical significance was set to *α* = 0.05. Size effects were reported with partial eta^2^ statistics (*η*_*p*_^*2*^).

## Results

**Fig 3:**
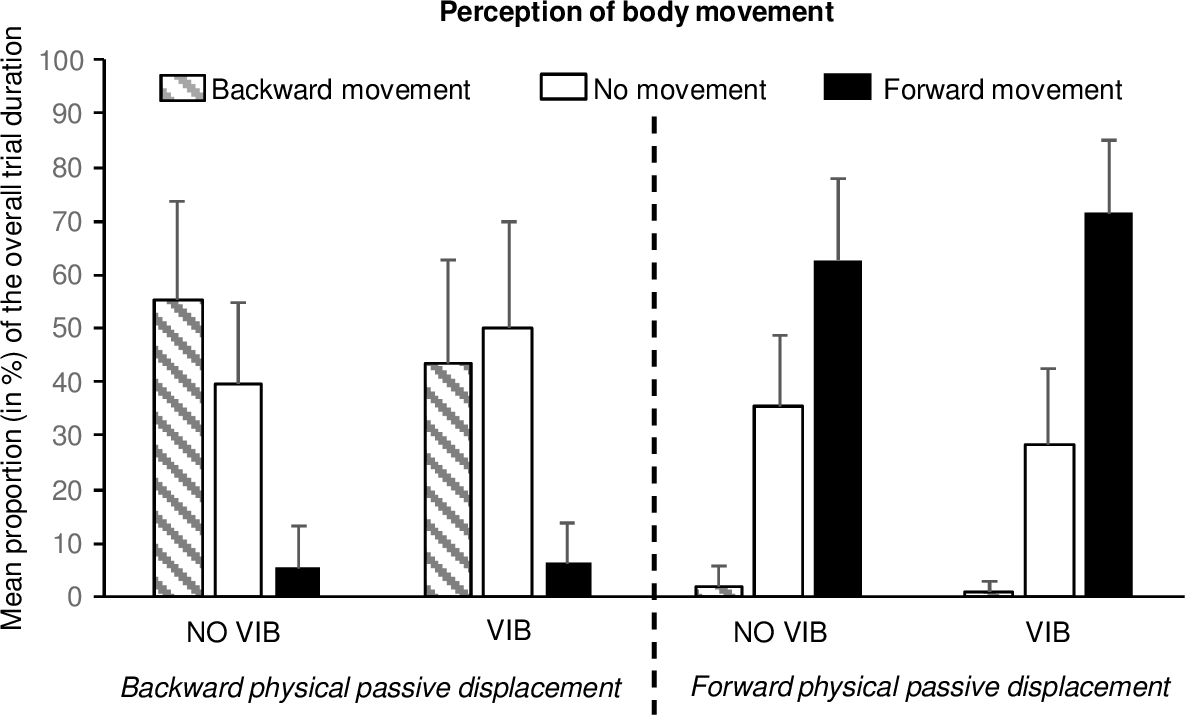
Participant’s response about their perception of body tilt movement as a function of the direction of physical passive displacement and the presence or absence of vibration. Mean proportion (in %) of the overall trial duration of each possible participant’s response (perception of a backward movement, perception of forward movement and no perception of movement, respectively indicated by hatched bars, black bars and white bars) for backward (left) and forward (right) physical passive displacement in the presence (VIB) or absence of Achilles tendon vibration (NO VIB). Error bars indicate the standard deviations.

**Fig 4:**
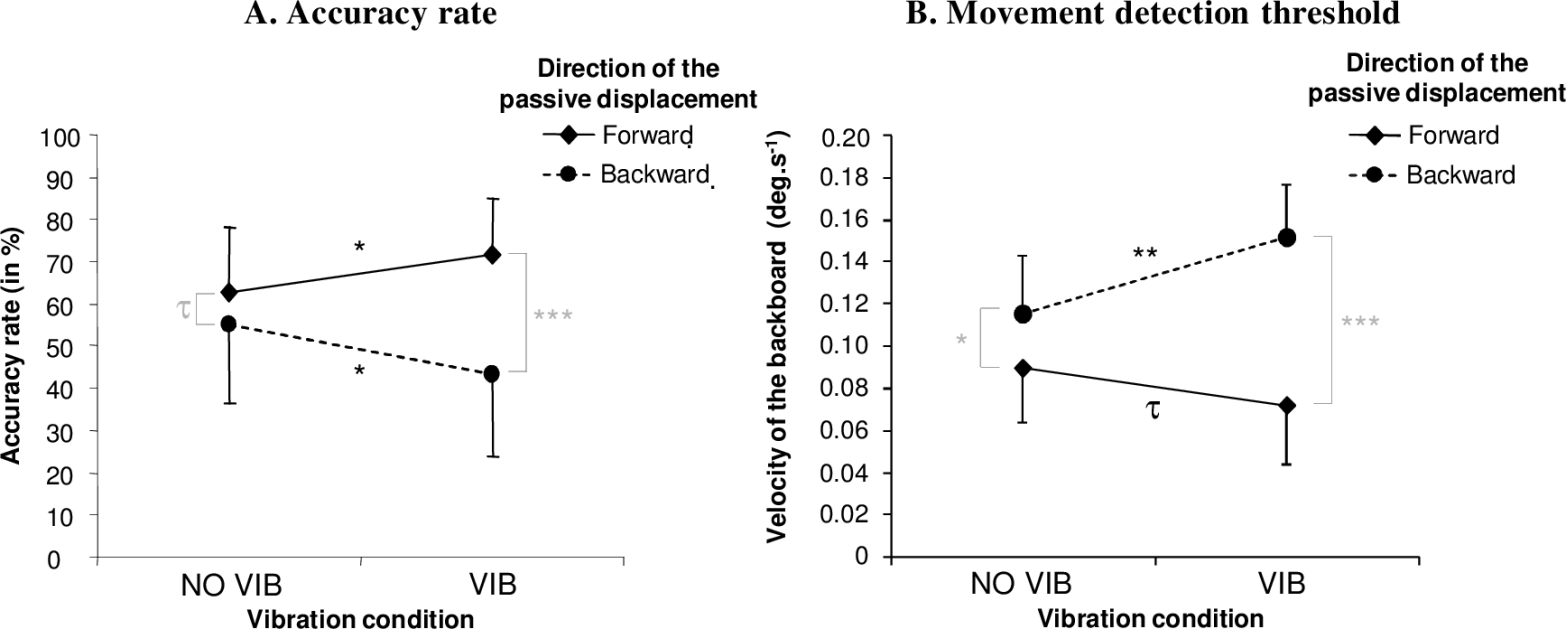
Accurate perception of the whole body tilt movement as a function of the direction of passive displacement and the presence or absence of vibration. (A) The mean accuracy rate, *i*.*e*. the mean proportion (in %) of the overall trial duration during which the passive displacement was correctly indicated by the participant, and (B) the mean movement detection threshold, *i*.*e*. the mean instantaneous angular velocity recorded at the time of the first accurate response maintained for at least 300 ms for forward displacement (rhombus symbols and solid lines) or backward displacement (dots and dashed lines) in the presence (VIB) or absence of Achilles tendon vibration (NO VIB). Error bars indicate the standard deviations. Asterisks and symbols indicate the *p*-level during NK pairwise comparisons: * p < 0.05; ** p < 0.01; *** p < 0.01; τ: p ≤ 0.10.

There were no differences in either the accuracy rate or the movement detection threshold as a function of the participant’s initial position (i.e. starting from either 0° or from a tilt of ±4 deg) in both the forward and backward conditions and when the VIB and NO VIB conditions were considered (p > 0.05 for all). Therefore, for the following analyses, data recorded for forward displacements from -4 deg to 0 deg and from 0 deg to +4 deg were pooled, as were data recorded for backward displacements from +4 deg to 0 deg and from 0 deg to -4 deg. Hence, only the direction of the passive displacement was assessed in the analyses presented below. Concerning the participant’s perception of their body movement, it should be noted that an accurate response (at least for a given period) has been provided in each condition and for all the participants, in almost all of the trials. Indeed, for the entire experiment, this was not the case for only 2 trials (of the participant “S05”) in Bw-NO VIB condition and for only 2 trials (of the participants “S03” and “S05”) in Bw-VIB condition. As indicated in Fig 3, both in NO VIB and VIB conditions, participant’s misperception about their (passive) body movement was much more frequently related to an absence of response (joystick released) than a reversed perception of the physical displacement. Descriptive results about accuracy rates and movement detection thresholds are presented in Fig 4.

### Accuracy rate

The ANOVA revealed a significant main effect of the direction of passive displacement [*F*(1,10) = 23.97; *p* < 0.001; *η*_*p*_^*2*^ = 0.71] and a significant interaction between the direction of passive displacement × vibration condition [*F*(1,10) = 14.06; *p* = 0.004; *η*_*p*_^*2*^ = 0.58] (Fig 4A). In fact, participants perceived a forward passive movement more accurately when vibration was applied (Fw-VIB: mean ± SD = 71.5% ± 13.6) than when the vibrators were off (Fw-NO-VIB: m = 62.6% ± 15.6; NK: *p* = 0.044). The opposite was observed during backward displacement: participants perceived backward movement less accurately when vibration was applied (Bw-VIB: m = 43.4% ± 19.4; Bw-NO VIB: m = 55.1% ± 18.7; NK: *p* = 0.014). In the absence of vibration, forward tilt tended to be more accurately perceived than backward tilt—although the difference did not quite reach statistical significance (NK: *p* = 0.083). The main effect of the vibration condition was not significant [*F*(1,10) = 0,07; *p* = 0.79].

### Movement detection threshold

The movement detection threshold (Fig 4B) was lower during forward displacement than during backward displacement, as indicated by the significant main effect of the direction of the passive displacement [*F*(1,10) = 63.00 *p* < 0.001; *η*_*p*_^*2*^ = 0.86]. More interestingly, as seen for the accuracy rate, a significant interaction between the direction of passive displacement × vibration condition was observed [*F*(1,10) = 14.15 *p* = 0.004; *η*_*p*_^*2*^ = 0.59]. Indeed, a greater velocity of displacement was required to trigger the perception of backward movements when vibration was applied (mean ± SD = 0.151 deg.s^-1^ ±0.026) than in its absence (m = 0.116 deg.s^-1^ ± 0.027; NK: *p* = 0.006). Conversely, the mean movement detection threshold tended to be lower in the Fw-VIB (m = 0.072 deg.s^-1^ ± 0.029) than in the Fw-NO VIB condition (m = 0.090 deg.s^-1^ ± 0.026). However, this effect of the vibration condition during forward passive displacement failed to reach statistical significance (*p* = 0.10). This could be partly explained by the fact that in the absence of vibration, forward tilts were perceived at a significantly lower velocity than backward tilts (NK: *p* = 0.028). Lastly, as for the accuracy rate, the main effect of the vibration condition was not significant [*F*(1,10) = 1.64; *p* = 0.23].

## Discussion

The objective of the present study was to investigate the contribution of proprioceptive input to whole-body movement perception. To this end, body movement perception was evaluated in participants placed in upright position, freed of balance constraints, and tilted passively backwards or forwards around the ankles via a backboard to which they were securely strapped. The backward and forward displacement could be combined with Achilles tendon vibration, which is known to simulate lengthening of the *triceps surae*. Our results showed that perception of forward movement was more accurate in the presence of Achilles tendon vibration than in its absence. The reverse was true for backward movement. Similar results were observed for the movement detection threshold.

Preventing postural responses (by freeing the participants of balance constraints) was a prerequisite for probing the kinesthetic perception of whole-body movement in a range of amplitudes compatible with the limits of stability. The low accuracy rate for movement perception during backward displacement combined with Achilles tendon vibration indicated that participants either failed to perceive the movement or perceived movement in the opposite (forward) direction more often in this situation; this is coherent with the simulated *triceps surae* stretch induced by Achilles tendon vibration. The fact that perception of both forward and backward movements was modulated by vibration (i.e. more accurate and less accurate perception of movement, respectively) indicates that the proprioceptive inflows relative to both real and simulated displacements were centrally integrated in order to update the postural schema in general and the kinesthetic perception of whole-body movement in particular. Previous studies about whole-body tilt have investigated rather the sense of position by demonstrating that simulating a forward tilt with Achilles tendon vibration leads to misperception of the postural orientation and verticality [31,33]. However, given that muscle-tendon vibration stimulates the primary endings in particular [17,18], it is often assumed that the resulting simulated displacement has a dynamic component. Our novel results confirm that muscle-tendon vibration modulates not only the sense of body position (as previously demonstrated) but also the sense of body movement. Therefore, by analogy with the known kinesthetic effects of vibration on body segments (notably the upper limbs), our present findings showed that this kind of stimulation also modulates whole-body movement perception.

Most studies of vibration-induced illusions have looked at perceptual and motor effects on a limb and more especially on the upper limb. In contrast, our present study focused on the effect on the body orientation perception. This raises the question of whether vibration’s effects on the upper limb are similar to its effects on the whole-body. Cordo et al. [20] investigated the interaction between (i) the illusory movements induced by vibrations of the *triceps brachii* and (ii) small (2–4 deg), slow (3 deg.s^-1^), passive rotations of the elbow. Their results showed that the illusory movement induced by vibration was enhanced, stopped or even reversed, depending on the direction of the passive joint rotation. Therefore, the direction of slow passive arm displacements was correctly perceived even when an incongruent kinesthetic illusion was elicited beforehand. Our results agree with this previous finding; during passive displacements, participants accurately perceived the true direction of the whole-body movement (at least for a given period of time within each trial), regardless of the congruence with the vibration-induced effect. Although artificial proprioceptive stimulation has an impact on whole-body movement perception, the imposed real displacement therefore predominates in the determination of the features of the perceived whole-body movement. One can reasonably assume that vibration stimulates mainly type Ia afferents [17,18], whereas slow, low-amplitude, passive displacement activates additional afferents (in particular those that respond to natural mechanical stimuli from type II or III afferents (see [20]). Therefore, for both the upper and lower limbs, the combination of real and simulated displacements leads to a complex integrative process allowing the construction of the movement percept on the basis of different somatosensory inputs. Lastly, one should note an important difference between the laws governing upper and lower limb motion perception. In fact, Cordo et al. [20] reported that the time needed to detect passive arm movement was similar in the presence and absence of vibration. In contrast, we found that the movement detection threshold (expressed as an angular velocity in our experiment) was significantly affected by the vibration condition. Therefore, the ability to detect the onset of an upper limb movement or a whole-body movement might be underpinned by different mechanisms for the integration of proprioceptive inputs.

The proportion of the overall trial duration during which the passive displacement was correctly perceived depended on the experimental condition (as expected). However, the mean accuracy rates, which did not reach 100% whatever the participant and the experimental condition, showed that the movement was never accurately perceived in its entirety. Indeed, movement perception is closely related to both displacement velocity and tilt amplitude. One might wonder what participants felt when they did not report an accurate response. Our results (Fig 3) showed that these periods of inaccurate response were more often related to an absence of response than to a body movement perception in the opposite direction of the physical passive displacement. The absence of response might be related for the participant either (1) to a perception of being static; (2) to a phase of uncertainty as to whether he/she is static or being moved; or, finally, (3) to a perception of being moved without knowing the direction of the displacement. It must be remembered that participants were required to indicate with their joystick the direction of the perceived movement. In that respect, the experiment was not designed to identify the meaning of the period during which no movement was reported. Further experiments would be required to understand precisely what participants actually feel when they do not respond while being objectively displaced.

In the present study, participants were blindfolded, and the passive tilts were implemented at a very low acceleration (i.e. below the semicircular canals’ detection threshold). Therefore, proprioceptive cues contributed predominantly to the perception of the whole-body tilt movement. However, other available sensory cues could have been used to perceive movements. In particular, multimodal integration, possibly including contributions from otoliths, interoceptors and/or tactile-somatosensory cues, might explain why the real tilts were always well detected—even in presence of an incongruent simulated displacement. In our experimental set-up, we tried to homogenize tactile-somesthetic cues induced respectively by the pressure of the straps during forward tilt and the backboard during backward tilt. In contrast, a contribution of the interoceptive cues (*e*.*g*. stomach-related interoceptive cues) during forward and backward displacements cannot be ruled out [34].

## Conclusions

In the present study, we looked at how simulating whole-body displacement by muscle-tendon vibration modulated the perception of real passive whole-body tilt movement around the ankle joints. In the absence of visual information and with otolith-generated information as the only vestibular cues, real whole-body tilt displacement could be detected effectively at an approximate minimum velocity of 0.1 deg.s^-1^. This ability was either enhanced or altered by the simulated displacement induced by vibration, depending on the congruence between the two signals. Our findings thus confirm that the various proprioceptive inputs of the lower limb contribute to both the sense of movement and the sense of position. The central integration of these proprioceptive inputs by the central nervous system has a prominent role in perception of whole-body orientation and thus in updating of the postural schema.

## Supporting information

S1 File Individual accuracy rates. Accuracy rate’s data for all the participants (S1 to S11) and mean accuracy rates (and SD) as a function of the direction of passive tilt [Forward (Fw) vs. Backward (Bw)] and the presence (VIB) or absence of vibration (NO-VIB).
(XLSX)

S2 File Individual movement detection thresholds. Movement detection threshold’s data for all the participants (S1 to S11) and mean movement detection thresholds (and SD) as a function of the direction of passive tilt [Forward (Fw) vs. Backward (Bw)] and the presence (VIB) or absence of vibration (NO-VIB).
(XLSX)
